# Patients knowledge attitudes and practices regarding superficial fungal infections suggest public health and patient education are warranted

**DOI:** 10.1038/s41598-025-98919-8

**Published:** 2025-04-29

**Authors:** Yan Ma, Wen Cen, Meiqing Duan, Jing Yang, Yan Wang, Liang Gao, Guizhi Miao, Wenli Feng

**Affiliations:** Department of Dermatology, The Second Clinic College of Shanxi Medical University, Second Hospital of Shanxi Medical University, Taiyuan, 030001 China

**Keywords:** Knowledge, Attitude, Practice, Treatment, Superficial fungal infections, Diseases, Medical research

## Abstract

Superficial fungal infections are common worldwide and significantly impact public health. Understanding patients’ knowledge, attitudes, and practices (KAP) regarding their treatment and prognosis is essential for addressing gaps in care. This cross-sectional study utilized a self-designed KAP questionnaire to assess 456 patients with superficial fungal infections between October 15, 2023, and March 15, 2024. Data analysis included descriptive statistics, logistic regression, and structural equation modeling. The mean scores for knowledge, attitudes, and practices were 14.85 ± 7.75, 27.01 ± 4.28, and 22.91 ± 4.17, respectively. Key findings revealed that 32% of patients were unsure about long-term treatment, 51% misunderstood corticosteroid use, and 69% believed medications could be stopped once symptoms improved. Significant demographic disparities in KAP scores were observed, highlighting the influence of gender, age, education, and income. Patients exhibit inadequate knowledge, predominantly negative attitudes, and suboptimal practices regarding superficial fungal infections. Targeted educational interventions are crucial for enhancing treatment adherence, addressing misconceptions, and reducing antifungal resistance.

**Supplementary Information:**

The online version contains supplementary material available at 10.1038/s41598-025-98919-8.

## Introduction

Superficial fungal infections rank among the most common infections worldwide, affecting nearly two billion people globally^1^. These infections predominantly occur in tropical regions where the hot and humid climate creates an ideal environment for fungal growth. The prevalence of fungal infections is on the rise, driven by factors such as the indiscriminate use of antibiotics, anticancer therapies, and the presence of immunodeficiency diseases like AIDS^2^. Fungal diseases annually result in approximately 1.6 million deaths due to invasive infections caused by various fungi, notably Cryptococcus, Candida, Aspergillus, and Pneumocystis. Remarkably, this mortality rate is comparable to that of tuberculosis^3^. Furthermore, the emergence of antifungal resistance is becoming a significant concern. This includes the escalation of azole-resistant *Aspergillus fumigatus* and the spread of the multidrug-resistant yeast *Candida auris*, posing challenges to treatment and control strategies^4,5^.

The theory of Knowledge, Attitude, Belief, and Practice (KAP) posits that knowledge forms the foundation for behavior change, while attitudes and beliefs serve as the driving forces behind this change^6^. According to this theory, the process of human behavior modification encompasses three sequential steps: acquiring knowledge, forming attitudes and beliefs, and then establishing practices or behaviors^7^. However, the acquisition of knowledge alone does not necessarily result in behavior change; it must first alter perceptions, which in turn can change behavior through this new understanding^8^.

Dermatophytes, the primary pathogens responsible for superficial fungal infections, affect 20–25% of humans and animals annually, highlighting the significant prevalence of these infections^9^. The treatment of some superficial fungal infections like tineapedis and cruris often requires continued medication for two weeks beyond clinical cure with topical agents, and more persistent cases may necessitate ongoing systemic therapy to eradicate pathogens. This extensive treatment regimen can lead to decreased patient compliance and presents substantial challenges in clinical practice^10,11^.

Given the broad impact of these infections, particularly in regions with high incidence rates and among vulnerable populations, it is essential to understand how patients perceive and manage their conditions. Identifying knowledge gaps, variations in attitudes, and disparities in practices allows for the development of targeted educational interventions aimed at enhancing patient compliance and implementing effective treatment strategies. Furthermore, the escalating trend of antifungal resistance underscores the necessity for patients to be well-informed about medication adherence and usage. This study, therefore, seeks to explore the KAP concerning treatment approaches and the prognosis of superficial fungal infections, aiming to devise interventions that significantly mitigate the spread and severity of these infections and reduce their global health impact.

## Methods

### Study design and participants

This cross-sectional study was conducted between October 15, 2023, and March 15, 2024, targeting patients with superficial fungal infections. Ethical approval was granted by the Ethics Committee and written informed consent was secured via an electronic questionnaire before data collection began, with participation limited to those who consented. The study inclusion criteria required participants to have clear consciousness, normal cognitive function, no communication barriers, and the ability to complete the questionnaire independently.

The questionnaire, accessible via a link and QR code provided by Questionnaire Star (https://www.wjx.cn/), was distributed in consultation rooms and via WeChat groups. To facilitate data collection, five research assistants, comprising laboratory instructors and postgraduate students in dermatology, participated in distributing and collecting the questionnaires after receiving standardized training for the project’s survey. These assistants were well-versed in the diagnosis and treatment of superficial fungal infections. Research assistants were available to help clarify any misunderstandings about the questionnaire content for participants. The research team rigorously checked all questionnaires for completeness, internal consistency, and logical coherence. Any questionnaire that was incomplete, not filled within the designated time limit, submitted from the same IP address, or containing logical errors was excluded from the study.

### Questionnaire

The questionnaire design was informed by existing guidelines and relevant literature. Following its initial development, the questionnaire underwent a preliminary review by two professional experts in dermatology to ensure content validity. A pilot study involving 44 participants was conducted to test the clarity and reliability of the instrument, resulting in minor modifications based on participant feedback. The pilot study demonstrated good internal consistency, with Cronbach’s alpha of 0.804. After data collection in the main study, the questionnaire’s internal consistency was re-evaluated, yielding a Cronbach’s alpha coefficient of 0.892. Additionally, the Kaiser-Meyer-Olkin (KMO) coefficient of 0.931 confirmed its structural validity, indicating the suitability of the data for factor analysis. However, the questionnaire was not externally validated against established tools, which limits its generalizability beyond the study population. Future studies should consider incorporating validated instruments for comparison or external validation to enhance methodological robustness. The final questionnaire consisted of four sections: demographic data (including gender, age, education level, occupation, etc.), and dimensions assessing knowledge, attitudes, and practices related to superficial fungal infections (appendix).

The knowledge dimension comprised 28 questions addressing fundamental aspects of superficial fungal infections, treatment options, and prognosis. Correct responses were awarded one point, while incorrect or unclear responses received zero points, resulting in a possible score range of 0–28 points. The attitude dimension consisted of nine questions that evaluated perceptions of disease severity and attitudes towards treatment and prognosis prevention. Responses were scored using a five-point Likert scale, ranging from extremely positive (5 points) to extremely negative (1 point), allowing for a total score between 9 and 45 points. The practice dimension included six questions about treatment compliance, diet, exercise, and personal hygiene, also scored on a five-point Likert scale from always (5 points) to never (1 point), with a potential total score ranging from 6 to 30 points. Adequate knowledge, positive attitudes, and proactive practices were defined as achieving scores above 70% of the maximum possible in each respective dimension^12^.

### Sample size calculation

The calculation of the sample size was performed using the following formula^13^:

$$n = \left( {Z_{{(1 - a/2)}} /d} \right)^{2} \times p\left( {1 - p} \right).$$

In this formula: n represents the sample plan size, p was assumed to be 0.5 to maximize the sample size, α, the Type I error rate, was set at 0.05, resulting in (Z_(1-α/2) = 1.96, δ, the standard error, was assumed to be 0.05.

Taking into account an anticipated questionnaire response rate of 90%, the final target was to collect at least 430 completed questionnaires.

#### Statistical analysis

Statistical analysis was conducted using SPSS 27.0 and AMOS 26.0 (IBM Corp., Armonk, N.Y., USA). Continuous variables were described using mean ± standard deviation (SD), and between-group comparisons were performed using t-tests or analysis of variance (ANOVA). Categorical variables were presented as n (%). Pearson correlation analysis was employed to assess the correlations between knowledge, attitude, and practice scores. Univariate and multivariate logistic regression were performed to explore the risk factors associated with K, A, and P. Univariate variables with *P* < 0.05 were enrolled in multivariate regression. Structural equation modeling (SEM) was utilized to explore the relationships between knowledge (K), attitude (A), and practice (P). Two-sided *p* < 0.05 were considered statistically significant in this study.

## Results

This study collected a total of 632 cases, of which 456 were deemed valid. The overall Cronbach’s alpha coefficient for the questionnaire was 0.892, demonstrating good internal consistency, as further evidenced by a Kaiser-Meyer-Olkin (KMO) coefficient of 0.931. The participant demographics included 229 females (50.22%), 222 individuals aged 20–30 years (48.68%), 296 with college or undergraduate education (64.91%), 147 with an average monthly household income below 3000 yuan (32.24%), 82 with pets in the household (17.98%), and 277 with fungal infections of the skin (60.75%), followed by fungal infections of the nails in 135 participants (29.61%). The mean knowledge, attitude, and practice scores were 14.85 ± 7.75 (possible range: 0–28), 27.01 ± 4.28 (possible range: 9–45), and 22.91 ± 4.17 (possible range: 6–30), respectively. Analyses of demographic characteristics found that the knowledge scores varied from patients with different gender (*P* < 0.001), age (*P* < 0.001), marital status (*P* < 0.001), education (*P* < 0.001), occupation (*P* < 0.001), and average monthly household income (*P* < 0.001). As for the attitude score, there were difference among patients with different gender (*P* < 0.001), age (*P* < 0.001), education (*P* < 0.001), occupation (*P* = 0.026). The difference of practice score was found among patients with different age (*P* = 0.005), education (*P* = 0.001), and average monthly household income (*P* = 0.001) (Table 1).

**Table 1:** Basic information of participants and KAP score.

Variables	*N*(%)	Knowledge, mean ± SD	*P*	Attitude, mean ± SD	*P*	Practice, mean ± SD	*P*
*N* = 456							
Total score		14.85 ± 7.75		27.01 ± 4.28		22.91 ± 4.17	
Gender			< 0.001		< 0.001		0.417
Male	227 (49.78)	13.31 ± 7.77		26.28 ± 4.37		22.71 ± 4.22	
Female	229 (50.22)	16.38 ± 7.43		27.74 ± 4.06		23.10 ± 4.13	
Age			< 0.001		< 0.001		0.005
Below 20 years old	45 (9.87)	10.38 ± 7.02		24.13 ± 4.85		21.04 ± 3.32	
20–30 years old	222 (48.68)	16.86 ± 7.36		27.27 ± 4.01		23.32 ± 4.41	
31–40 years old	90 (19.74)	13.08 ± 7.95		27.11 ± 4.40		22.78 ± 4.19	
41–50 years old	64 (14.04)	15.08 ± 6.89		28.30 ± 3.34		22.92 ± 3.02	
Above 50 years old	35 (7.68)	12.00 ± 8.00		26.43 ± 4.85		22.94 ± 4.84	
Marital status			< 0.001		0.251		0.311
Married	205 (44.96)	13.32 ± 7.66		27.28 ± 4.25		22.65 ± 4.20	
Unmarried	251 (55.04)	16.10 ± 7.60		26.79 ± 4.29		23.12 ± 4.14	
Education			< 0.001		< 0.001		0.001
High school and below	121 (26.54)	9.90 ± 7.18		25.39 ± 4.61		21.89 ± 3.90	
College /undergraduate	296 (64.91)	16.38 ± 7.16		27.52 ± 4.08		23.36 ± 4.33	
Master’s degree and above	39 (8.55)	18.59 ± 6.87		28.18 ± 3.27		22.56 ± 3.16	
Occupation			< 0.001		0.026		0.154
White-collar/Corporate employee	98 (21.49)	14.18 ± 7.34		27.09 ± 4.22		22.77 ± 4.11	
Medical personnel	53 (11.62)	19.81 ± 5.73		28.23 ± 3.27		22.79 ± 4.21	
Manual laborer or primarily engaged in physical work	59 (12.94)	10.20 ± 7.12		25.95 ± 4.47		21.76 ± 4.93	
Other	246 (53.95)	15.16 ± 7.77		26.97 ± 4.39		23.26 ± 3.96	
Average monthly household income			< 0.001		0.067		0.001
< 3000	147 (32.24)	17.06 ± 7.29		27.72 ± 3.59		23.45 ± 3.82	
3000–5000	94 (20.61)	13.37 ± 7.21		27.10 ± 4.27		22.39 ± 4.02	
5000–10,000	67 (14.69)	13.78 ± 8.10		27.16 ± 4.45		21.36 ± 4.61	
> 10,000	31 (6.8)	14.65 ± 8.37		26.90 ± 4.28		24.65 ± 3.15	
Prefer not to disclose	117 (25.66)	13.93 ± 7.87		25.99 ± 4.81		23.06 ± 4.41	
Presence of Pets in Household			0.907		0.536		0.800
Yes	82 (17.98)	15.02 ± 7.10		27.20 ± 4.32		23.20 ± 3.81	
No	374 (82.02)	14.81 ± 7.89		26.97 ± 4.27		22.84 ± 4.25	
Affected Areas of Fungal Infection (multiple choices)							
Hair	78 (17.11)	16.15 ± 7.76		26.77 ± 4.52		23.51 ± 4.74	
Skin	277 (60.75)	14.60 ± 7.72		27.10 ± 4.21		22.86 ± 4.12	
Nails	135 (29.61)	15.28 ± 7.43		27.24 ± 4.17		22.77 ± 4.06	
Mucous membranes	30 (6.58)	17.13 ± 7.38		26.60 ± 4.21		23.17 ± 3.27	

**Fig. 1:**
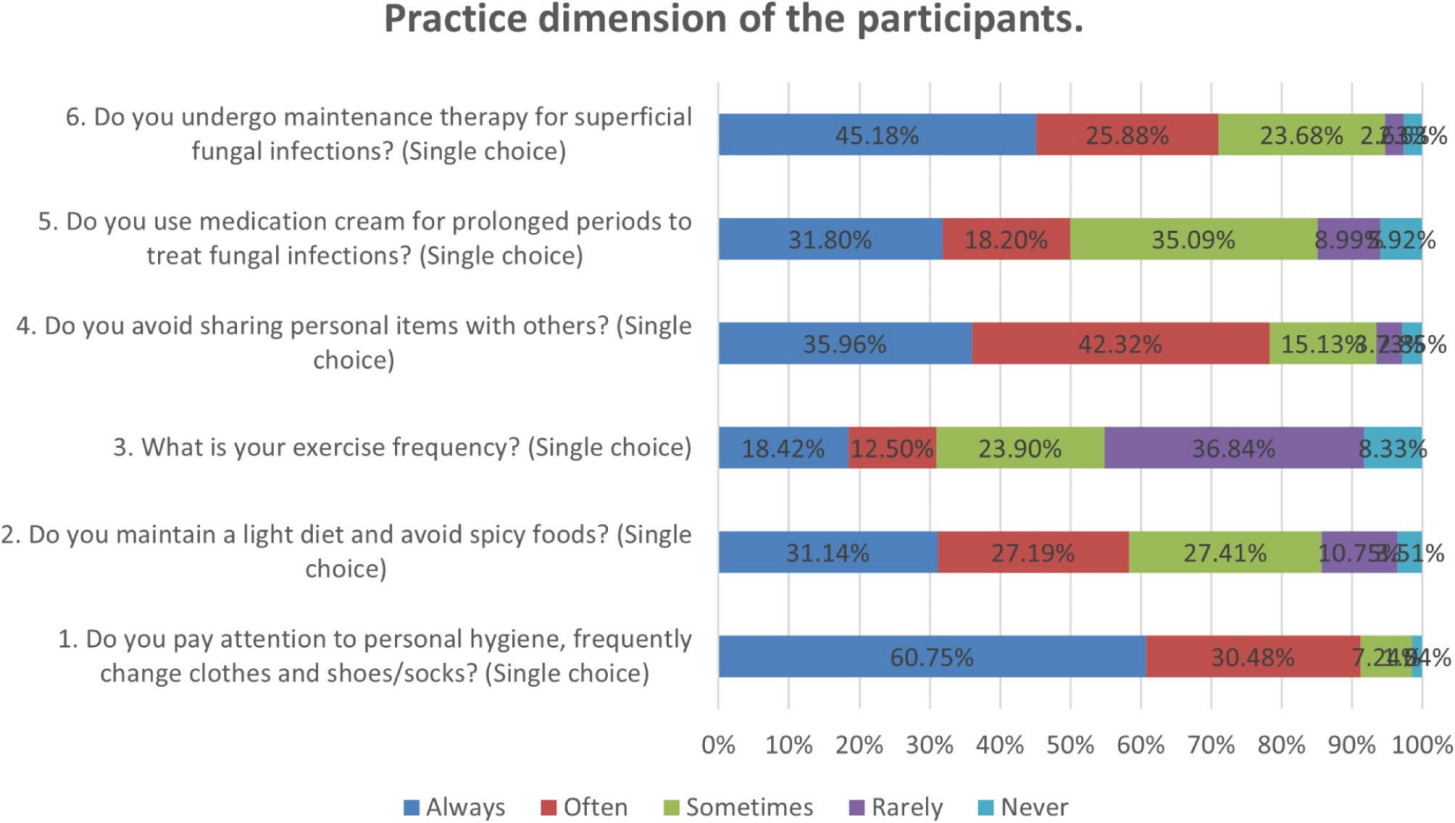
Percentage distribution of options per question for attitude and practice dimensions.

Responses to the knowledge dimension revealed notable misconceptions. For example, 32.24% were unclear about the necessity for long-term treatment and maintenance therapy (K12), 51.32% misunderstood the appropriateness of corticosteroid use (K7), 36.84% were unaware of the contagious nature of fungal infections (K10), and 69.08% incorrectly believed that medication can be discontinued once symptoms subside (K11). These findings are visually summarized in Fig. 1a, and detailed data are available in Table S1.

Responses to the attitude dimension showed that 34.65% strongly agreed that superficial fungal infections are common (A1), 5.92% strongly agreed and 5.7% agreed that ‘athlete’s foot’ cannot be cured, so it doesn’t matter whether it is cured or not (A7), 5.26% strongly agreed and 13.82% agreed that such infections will heal on their own (A4), and 10.09% totally disagreed that superficial fungal infections are difficult to cure (A6). It was clear that some of the participants had not taken the infection seriously enough and needed to be encouraged to take a positive attitude towards this infection (Fig. 1b).

When it comes to related practices, 36.84% basically do not exercise and 8.33% do not exercise at all (P3). At the same time, 10.75% basically did not and 3.51% not at all performed a light diet and avoided spices (P2) (Fig. 1c).

**Fig. 2:**
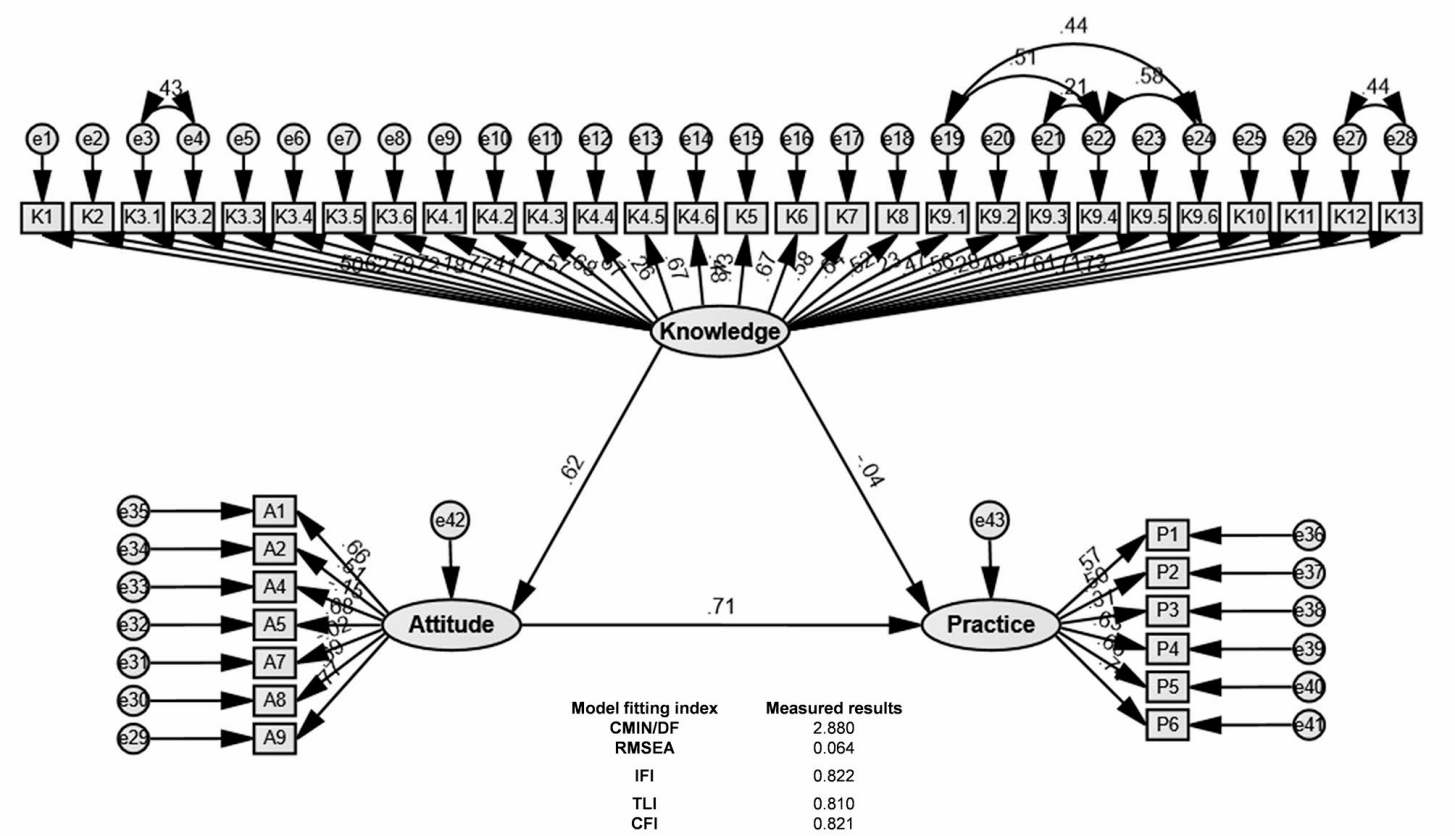
The Structural Equation Model (SEM). Rectangle shows observed variables, ellipses indicate potential variables, and circles represent residual terms.

In the correlation analysis, significant positive correlations were found between knowledge and attitude (*r* = 0.470, *P* = 0.002), knowledge and practice (*r* = 0.276, *P* < 0.001), and attitude and practice (*r* = 0.355, *P* < 0.001), respectively (Table 2).The results of SEM showed that knowledge directly affected attitude (β = 0.619, *P* = 0.007), attitude (β = 0.712, *P* = 0.014) directly affected practice, and knowledge indirectly affected practice through attitude (β = 0.440, *P* < 0.009) (Table 3; Fig. 2).

**Table 2:** Correlation analysis of KAP scores.

	Knowledge	Attitude	Practice
Knowledge	1		
Attitude	0.470 (*P* < 0.001)	1	
Practice	0.276 (*P* < 0.001)	0.355 (*P* < 0.001)	1

**Table 3:** Bootstrap analysis of mediating effect significance test for the final mode.

			Standardized total effects		Standardized direct effects		Standardized indirect effects	
			Beta (95%CI)	*P*	Beta (95%CI)	*P*	Beta (95%CI)	*P*
Attitude	<---	Knowledge	0.619 (0.537-0.700)	0.007	0.619 (0.537-0.700)	0.007		
Practice	<---	Attitude	0.712 (0.508–0.853)	0.014	0.712 (0.508–0.853)	0.014		
Practice	<---	Knowledge	0.405 (0.299–0.489)	0.018	-0.035 (-0.178-0.112)	0.617	0.440 (0.326–0.588)	0.009

Multivariate logistic regression showed that attitude score (OR = 1.197, 95% CI: [1.120–1.278], *P* < 0.001), aged 20–30 years old (OR = 3.233, 95% CI: [1.291–8.097], *P* = 0.012), aged 41–50 years old (OR = 2.883, 95% CI: [1.036–8.018], *P* = 0.043), aged more than 50 years old (OR = 3.616, 95% CI: [1.221–10.708], *P* = 0.020), with Master’s degree and above (OR = 0.356, 95% CI: [0.134–0.945], *P* = 0.038), with average monthly household income of 5000–10,000 yuan (OR = 0.455, 95% CI: [0.216–0.957], *P* = 0.038), and with average monthly household income of more than 10,000 yuan(OR = 3.356, 95% CI: [1.222–9.214], *P* = 0.019) were independently associated with practice (Table S2).

## Discussion

Patients with superficial fungal infections exhibit insufficient knowledge, predominantly negative attitudes, and suboptimal practices concerning their treatment and prognosis. This reflects a significant gap in patient education and engagement. Addressing these gaps through targeted educational initiatives is essential, as misconceptions about treatment protocols and inadequate adherence to recommended practices can compromise treatment outcomes and contribute to antifungal resistance. Tailored interventions must emphasize the importance of completing prescribed treatment courses and avoiding premature discontinuation of medications to prevent recurrence and resistance. Given the growing global burden of superficial fungal infections and the alarming rise in antifungal resistance, addressing these gaps is essential for developing effective public health strategies. The findings from this study highlight the need for policies that promote equitable access to education and treatment for fungal infections. Integrating fungal infection education into primary care settings and public health campaigns could reduce disparities in knowledge and adherence, particularly among low-income groups who may face barriers to accessing medical care. These initiatives would ensure that all patients, regardless of socioeconomic status, receive adequate information and resources to manage their conditions effectively.

The findings from this study underscore significant disparities in the KAP concerning the treatment and prognosis of superficial fungal infections across different demographic groups, supported multivariate logistic regression analyses. Notably, females exhibited superior knowledge and attitudes compared to males. This gender difference in health literacy and engagement is consistent with previous studies indicating that women are generally more proactive about seeking health information and adhering to treatment recommendations^14^. Age was another critical determinant, with younger adults (20–30 years) demonstrating higher knowledge and practice scores. This could be attributed to younger individuals’ better access to and familiarity with health information through digital media, a trend observed in broader health communication research^15,16^.

Educational attainment and occupation significantly influenced KAP outcomes. Participants with higher education levels, particularly those with Master’s degrees, scored highest in knowledge and attitudes. This finding aligns with literature suggesting that higher educational levels are associated with improved health literacy, which in turn facilitates better understanding and engagement with health care practices^17,18^. Similarly, medical personnel had the highest knowledge scores, likely due to their professional background and access to up-to-date medical information. These results are supported by the multivariate logistic regression, indicating a strong, independent association between higher education and professional occupation with better practices. Income level also played a significant role, with lower-income individuals surprisingly showing higher knowledge and practice scores. This counterintuitive result may reflect the necessity-driven engagement in self-care due to limited access to healthcare services^19,20^. This aspect was further confirmed by logistic regression analysis, highlighting a significant association between lower income and the likelihood of adopting effective practices.

Correlation analysis and SEM revealed significant positive relationships between knowledge and attitudes, and both directly influencing practices. The cascading effect observed in SEM suggests that improving health knowledge can lead to better practices indirectly by fostering positive attitudes. This underscores the importance of addressing both cognitive and attitudinal factors in designing interventions, as strengthening knowledge alone may not be sufficient without corresponding attitudinal changes. This cascading effect of KAP components suggests that improving knowledge can have a direct and indirect positive impact on practices through attitudes, an observation consistent with health behavior theories^11,21^.

The results from this study highlight varied levels of knowledge, attitudes, and practices among patients regarding the treatment and management of superficial fungal infections. In the knowledge dimension, significant gaps were noted particularly in understanding the applicability of treatments like topical corticosteroids and the contagious nature of these infections. Many patients were uncertain about whether conditions like diarrhea are symptoms of fungal infections or if superficial fungal infections can lead to severe complications if untreated. Similarly, attitudes towards the severity and treatability of fungal infections varied, with a considerable number of participants underestimating the impact of lifestyle changes on managing these infections. To address these gaps, public health campaigns and primary care programs should include educational initiatives that emphasize the risks of incomplete treatment, proper medication use, and the role of preventive behaviors such as maintaining personal hygiene and a balanced lifestyle. These efforts could leverage digital platforms, community workshops, and clinician-led counseling to ensure comprehensive coverage and accessibility for diverse populations. The practice dimension also showed discrepancies; while a high percentage of patients adhere to personal hygiene, the practice of maintaining a light diet and regular exercise was less prevalent. To enhance the treatment and management of superficial fungal infections, our findings point to the need for targeted interventions aimed at specific demographic groups who exhibit significant knowledge gaps and suboptimal practices. These interventions should also align with global public health objectives, such as mitigating antifungal resistance through improved treatment adherence and reducing disease transmission. For instance, campaigns targeting men could emphasize the importance of sustained treatment and hygiene practices, while also leveraging community engagement platforms to raise awareness about the broader implications of antifungal resistance. Such initiatives contribute to a more patient-centered healthcare approach, which is increasingly recognized as vital in addressing global health challenges^22,23^. For younger adults, especially those between 20 and 30 years who demonstrated better knowledge and practices, digital engagement could be intensified. Online platforms are ideal for circulating animated videos and infographics that explain symptoms, risks, and treatments in a format that appeals to this age group. Educational programs must also address the varying levels of understanding across different education levels and occupations. For individuals with lower educational attainment or in non-medical occupations who may lack access to reliable health information, community-based workshops could be invaluable. These workshops can be held in community centers and staffed by medical professionals. They should focus on practical demonstrations on how to correctly use medications, the importance of completing treatment courses, and lifestyle modifications that can prevent the exacerbation of symptoms^24–26^.

Given the influence of income on treatment practices, with lower-income individuals showing surprisingly better knowledge and practices, it is crucial to ensure that these groups continue to have access to affordable healthcare services and medications. Subsidies for antifungal medications and educational outreach targeting low-income groups could bridge disparities and support better adherence. Additionally, integrating fungal infection education into primary healthcare settings can ensure consistent messaging and long-term engagement. In addition to addressing these specific demographic needs, general strategies should also be employed to improve overall patient outcomes. For example, all patient educational materials should highlight the importance of not only treating the infection with the correct medications but also adhering to preventive measures post-treatment to avoid recurrence^27,28^.

This study has several limitations worth noting. First, the self-reported nature of the KAP questionnaire may introduce response bias, as participants might provide socially desirable answers rather than accurate reflections of their knowledge and practices. Second, while the questionnaire demonstrated good internal consistency and structural validity, it was not externally validated against established tools, limiting its application beyond the study population. Future studies should aim to validate the instrument against widely accepted scales to improve its reliability and applicability across diverse settings. Third, the sample was drawn from a single hospital, restricting the generalizability of the findings. Multicenter studies across different regions are recommended to enhance external validity and capture broader population dynamics. Finally, the cross-sectional design of the study precludes the establishment of causality between observed associations. The exploratory nature of this study should be emphasized, and the findings should be interpreted with caution, as associations cannot be presumed to indicate causal relationships.

In conclusion, patients with superficial fungal infections exhibit inadequate knowledge, predominantly negative attitudes, and moderately proactive practices concerning their treatment options and prognosis. Clinical programs should focus on educational interventions to enhance knowledge and shift attitudes, thereby improving treatment practices among these patients. This study highlights the urgent need for targeted educational strategies and public health policies to address these knowledge gaps and improve adherence to antifungal treatment regimens. By enhancing patient understanding and correcting misconceptions, these interventions can reduce the burden of antifungal resistance and improve long-term health outcomes. Furthermore, integrating the findings into global health initiatives, such as promoting equitable access to affordable antifungal treatments and resources, could contribute to broader public health improvements. Future research should prioritize multicenter studies and externally validated instruments to enhance methodological rigor and generalizability. Additionally, the findings underline the importance of addressing social determinants of health in the management of fungal infections, emphasizing the role of tailored, patient-centered care in fostering sustainable treatment practices.

## Electronic supplementary material

Below is the link to the electronic supplementary material.

Supplementary Material 1

Supplementary Material 2

## Data Availability

All data generated or analyzed during this study are included in this published article.
