# Supplementary material for: Patients knowledge attitudes and practices regarding superficial fungal infections suggest public health and patient education are warranted
Source: Sci Rep. 2025 Apr 29;15:15112. doi: 10.1038/s41598-025-98919-8 (PMC12041239; doi:10.1038/s41598-025-98919-8)
Supplement: Supplementary file 1 — Supplementary Material 1 [file 41598_2025_98919_MOESM1_ESM.docx]

Questionnaire Code:

| Dear Friend,  We are researchers from Hospital, and we sincerely invite you to participate in our research project. This study aims to understand the Knowledge, Attitudes, and Practices (KAP) of patients with superficial fungal infections towards treatment methods and prognosis.  1.Please complete the questionnaire. There are no right or wrong answers; you only need to fill in according to your actual situation. If you have any questions during the answering process, please feel free to ask us. After completion, please submit the questionnaire promptly.  2.This study is a simple questionnaire survey that will not cause harm to your physical or psychological condition. However, it will involve some privacy issues such as your gender, age, etc. Rest assured that we will strictly protect your privacy and will not disclose your information. Please feel free to fill in.  3.As a participant, you can always know the information and progress related to this study. If you decide to withdraw from the study, please inform us, and your data will not be included in the study results.  Finally, we sincerely thank you for taking the time out of your busy schedule to support our scientific research!  □I have been informed and consent to the use of the collected data for scientific research.  Date of Participation: Year Month Day |
| --- |

**A KAP Study on the Treatment Methods and Prognosis of Superficial Fungal Infections Among Patients**

**Part One: Sociodemographic Data**

1. Gender：
2. Male
3. Female
4. Age：
5. Below 20 years old
6. 20~30 years old
7. 31~40 years old
8. 41~50 years old
9. Above 50 years old
10. Marital status：
    - 1. Married
      2. Unmarried
      3. Widowed
      4. Divorced
11. Education：
12. Primary school and below
13. Junior high school
14. High school/technical school
15. Bachelor’s /associate degree
16. Master’s degree and above
17. Occupation：
    1. White-collar/Corporate employee
    2. Educator
    3. Medical personnel
    4. Manual laborer or primarily engaged in physical work
    5. Homemaker
    6. Retired
    7. Other (please specify)
18. Monthly Income (RMB):
    1. ＜3000
    2. 3000~5000
    3. 5000~10000
    4. ＞10000
    5. Prefer not to disclose
19. Affected Areas of Fungal Infection (multiple choices):
    1. Hair
    2. Skin
    3. Nails
    4. Mucous membranes
20. Do you have pets at home?
    1. Yes
    2. No

**Part Two**

**Survey on the Knowledge of Superficial Fungal Infection Patients Regarding Treatment Methods and Prognosis**

1. Tinea corporis, a superficial fungal infection, is commonly known as "ringworm"? (Single choice)
   1. The statement is correct
   2. The statement is incorrect
   3. Uncertain
2. Which of the following fungi does not cause fungal infections? (Single choice)
   1. Dermatophyte
   2. Candida
   3. Escherichia coli
   4. Mold
   5. Uncertain
3. Are the following diseases caused by superficial fungal infections?
   1. Athlete's foot (Tinea pedis) --------------- (a. Yes b. No c. Uncertain)
   2. Jock itch (Tinea cruris) --------------------------------- (a. Yes b. No c. Uncertain)
   3. Tinea versicolor------------------------------- (a. Yes b. No c. Uncertain)
   4. Nail fungus (Onychomycosis) --------------- (a. Yes b. No c. Uncertain)
   5. Eczema -------------------------------------- (a. Yes b. No c. Uncertain)
   6. Scalp ringworm (Tinea capitis) --------------- (a. Yes b. No c. Uncertain)
4. Do superficial fungal infections present with the following symptoms?
   1. Skin blisters ------------------------------- (a. Yes b. No c. Uncertain)
   2. Skin itching -------------------------------- (a. Yes b. No c. Uncertain）
   3. Hair loss ----------------------------------- (a. Yes b. No c. Uncertain)
   4. Diarrhea ------------------------------------ (a. Yes b. No c. Uncertain)
   5. Skin peeling -------------------------------- (a. Yes b. No c. Uncertain)
   6. Oral ulcers --------------------------------- (a. Yes b. No c. Uncertain)
5. If left untreated, can superficial fungal infections lead to severe skin erosion and ulceration? (Single choice)
6. Yes
7. No
8. Uncertain
9. If left untreated, can superficial fungal infections lead to a weakened immune system? (Single choice)
10. Yes
11. No
12. Uncertain
13. Can topical corticosteroid creams be used to treat superficial fungal infections? (Single choice)
14. Yes
15. No
16. Uncertain
17. Can oral antifungal medications treat superficial fungal infections? (Single choice)
18. Yes
19. No
20. Uncertain
21. Do the following habits help improve superficial fungal infections?
    1. Paying attention to personal hygiene --------------- (a. Yes b. No c. Uncertain)
    2. Wiping the affected area with alcohol ---------------- (a. Yes b. No c. Uncertain)
    3. Keeping the affected area dry ----------------------- (a. Yes b. No c. Uncertain)
    4. Increasing exercise ----------------------------------- (a. Yes b. No c. Uncertain)
    5. Sweat therapy ---------------------------------------- (a. Yes b. No c. Uncertain)
    6. Eating more vegetables ------------------------------- (a. Yes b. No c. Uncertain)
22. Are superficial fungal infections contagious? (Single choice)
23. Yes
24. No
25. Uncertain
26. Can medication cream be stopped as long as the affected area is not itchy? (Single choice)
27. Yes
28. No
29. Uncertain
30. Is it correct that superficial fungal infections have a longer treatment period and require maintenance therapy? (Single choice)
31. The statement is correct
32. The statement is incorrect
33. Uncertain
34. Do superficial fungal infections recur? (Single choice)
35. Yes
36. No
37. Uncertain

**Part Three**

**Survey on the Attitudes of Superficial Fungal Infection Patients Towards Treatment Methods and Prognosis**

1. Do you think superficial fungal infections are common? (Single choice)

a. Very common

b. Common

c. Average

d. Not very common

e. Rare

1. Do fungal infections such as athlete's foot, ringworm, and jock itch affect your quality of life? (Single choice)
2. Very much
3. Somewhat
4. Neutral
5. Not much
6. Not at all
7. Do you feel embarrassed about having fungal infections such as athlete's foot? (Single choice)
8. Very embarrassed
9. Somewhat embarrassed
10. Neutral
11. Not very embarrassed
12. Not embarrassed
13. Do you believe that superficial fungal infections will heal on their own? (Single choice)
14. Strongly agree
15. Agree
16. Neutral
17. Disagree
18. Strongly disagree
19. How do you think lifestyle habits affect fungal infections? (Single choice)
20. Significant impact
21. Some impact
22. Neutral
23. Little impact
24. No impact
25. Do you think superficial fungal infections are difficult to cure? (Single choice)
26. Strongly agree
27. Agree
28. Neutral
29. Disagree
30. Strongly disagree
31. Do you believe that "athlete's foot" cannot be cured, so it doesn't matter whether it's treated or not? (Single choice)
32. Strongly agree
33. Agree
34. Neutral
35. Disagree
36. Strongly disagree
37. Can failure to treat fungi lead to other more serious diseases? (Single choice)
38. Strongly agree
39. Agree
40. Neutral
41. Disagree
42. Strongly disagree
43. After the fungal infection is cured, do you think continued prevention is necessary? (Single choice)
44. Very necessary
45. Necessary
46. Neutral
47. Not very necessary
48. Not necessary

**Part Four**

**Survey on the Practices of Superficial Fungal Infection Patients Towards Treatment Methods and Prognosis**

1. Do you pay attention to personal hygiene, such as changing clothes and socks frequently? (Single choice)
   1. Always
   2. Often
   3. Occasionally
   4. Rarely
   5. Never
2. Do you maintain a bland diet and avoid spicy food? (Single choice)
   1. Always
   2. Often
   3. Occasionally
   4. Rarely
   5. Never
3. How often do you exercise? (Single choice)
4. Almost every day
5. 3-5 times a week
6. 1-2 times a week
7. Rarely
8. Never
9. Do you avoid sharing personal items with others? (Single choice)
10. Always strictly avoid, constantly remind myself
11. Mostly avoid in most cases
12. Not sure, depends on the situation
13. Often forget, can't avoid sharing items
14. Don't consciously avoid sharing items
15. Do you use medicated cream for a long time to treat fungal infections? (Single choice)
16. Always, I want to completely cure the infection
17. Yes, long-term use may yield better results
18. Not sure, depends on the situation
19. Not likely, I often forget to use it
20. Never, I believe there are side effects
21. Do you undergo maintenance treatment for superficial fungal infections? (Single choice)
22. Always, I want to completely cure the infection
23. Yes, maintenance treatment may yield better results
24. Not sure, depends on the situation
25. Not likely, I often forget to use it
26. Never, I believe there are side effects
