# Supplementary material for: Patients knowledge attitudes and practices regarding superficial fungal infections suggest public health and patient education are warranted
Source: Sci Rep. 2025 Apr 29;15:15112. doi: 10.1038/s41598-025-98919-8 (PMC12041239; doi:10.1038/s41598-025-98919-8)
Supplement: Supplementary file 2 — Supplementary Material 2 [file 41598_2025_98919_MOESM2_ESM.docx]

Table S1. Knowledge dimension of the participants.

|  | Correct options | **N（%）** | | |
| --- | --- | --- | --- | --- |
|  |  | **Yes** | **No** | **Uncertain** |
| **1.Tineacorporis, a superficial fungal infection, is commonly known as "ringworm"? (Single choice)** | Yes | 216 (47.37) | 29 (6.36) | 211 (46.27) |
| **3.Which of the following diseases are caused by superficial fungal infections?** |  |  |  |  |
| **（1）Athlete's foot** | Yes | 285 (62.5) | 20 (4.39) | 151 (33.11) |
| **（2）Jock itch** | Yes | 234 (51.32) | 35 (7.68) | 187 (41.01) |
| **（3）Tineaversicolor** | No | 171 (37.5) | 87 (19.08) | 198 (43.42) |
| **（4）Nail fungus** | Yes | 227 (49.78) | 33 (7.24) | 196 (42.98) |
| **（5）Eczema** | No | 126 (27.63) | 127 (27.85) | 203 (44.52) |
| **（6）Scalp ringworm** | Yes | 233 (51.1) | 36 (7.89) | 187 (41.01) |
| **4.Does superficial fungal infection present with the following symptoms?** |  |  |  |  |
| **（1）Appearance of blisters on the skin** | Yes | 194 (42.54) | 85 (18.64) | 177 (38.82) |
| **（2）Skin itching** | Yes | 330 (72.37) | 23 (5.04) | 103 (22.59) |
| **（3）Hair loss** | Yes | 229 (50.22) | 75 (16.45) | 152 (33.33) |
| **（4）Diarrhea** | No | 65 (14.25) | 198 (43.42) | 193 (42.32) |
| **（5）Skin peeling** | Yes | 300 (65.79) | 37 (8.11) | 119 (26.1) |
| **（6）Oral ulcers** | Yes | 147 (32.24) | 136 (29.82) | 173 (37.94) |
| **5.If left untreated, can superficial fungal infections lead to severe skin erosion and ulceration? (Single choice)** | Yes | 266 (58.33) | 28 (6.14) | 162 (35.53) |
| **6.If left untreated, can superficial fungal infections lead to a weakened immune system? (Single choice)** | Yes | 254 (55.7) | 35 (7.68) | 167 (36.62) |
| **7.Can topical corticosteroid creams be used to treat superficial fungal infections? (Single choice)** | Yes | 169 (37.06) | 53 (11.62) | 234 (51.32) |
| **8.Can oral antifungal medications treat superficial fungal infections? (Single choice)** | Yes | 195 (42.76) | 44 (9.65) | 217 (47.59) |
| **9.Which of the following habits can help improve superficial fungal infections?** |  |  |  |  |
| **（1）Paying attention to personal hygiene** | Yes | 401 (87.94) | 3 (0.66) | 52 (11.4) |
| **（2）Wiping the affected area with alcohol** | No | 216 (47.37) | 101 (22.15) | 139 (30.48) |
| **（3）Keeping the affected area dry** | Yes | 348 (76.32) | 20 (4.39) | 88 (19.3) |
| **（4）Increasing exercise** | Yes | 382 (83.77) | 13 (2.85) | 61 (13.38) |
| **（5）Sweat therapy** | No | 128 (28.07) | 123 (26.97) | 205 (44.96) |
| **（6）Eating more vegetables** | Yes | 382 (83.77) | 13 (2.85) | 61 (13.38) |
| **10. Are superficial fungal infections contagious? (Single choice)** | Yes | 248 (54.39) | 40 (8.77) | 168 (36.84) |
| **11. Can medication cream be stopped as long as the affected area is not itchy? (Single choice)** | No | 34 (7.46) | 315 (69.08) | 107 (23.46) |
| **12. Is superficial fungal infection treatment usually of longer duration and requires maintenance therapy? (Single choice)** | Yes | 299 (65.57) | 10 (2.19) | 147 (32.24) |
| **13. Do superficial fungal infections recur? (Single choice)** | Yes | 291 (63.82) | 9 (1.97) | 156 (34.21) |
| **2.Which of the following fungi does not cause fungal infections? (Single choice)** |  |  |  |  |
| **Tineacorporis** |  | 23 (5.04) |  |  |
| **Candida albicans** |  | 7 (1.54) |  |  |
| **Escherichia coli (Correct option)** |  | 191 (41.89) |  |  |
| **Mold** |  | 15 (3.29) |  |  |
| **Uncertain** |  | 220 (48.25) |  |  |

Table S2. Factors of practice based univariable and multivariable logistic regression.

| Practice cutoff=6+（30-6）*0.7=22.8 | Univariate logistic regression | | Multivariate logistic regression | |
| --- | --- | --- | --- | --- |
|  | OR (95%CI) | P | OR (95%CI) | P |
| **Knowledge total score** | 1.064 (1.038-1.092) | **<0.001** | 1.016 (0.982-1.051) | 0.356 |
| **Attitude total score** | 1.200 (1.135-1.267) | **<0.001** | 1.197 (1.120-1.278) | **<0.001** |
| **Age** |  |  |  |  |
| Below 20 years old | ref |  | ref |  |
| 20-30 years old | 3.437 (1.623-7.280) | **0.001** | 3.233 (1.291-8.097) | **0.012** |
| 31-40 years old | 2.026 (0.890-4.616) | 0.093 | 2.272 (0.836-6.174) | 0.108 |
| 41-50 years old | 3.288 (1.396-7.746) | **0.006** | 2.883 (1.036-8.018) | **0.043** |
| Above 50 years old | 3.306 (1.258-8.686) | **0.015** | 3.616 (1.221-10.708) | **0.020** |
| **Education** |  |  |  |  |
| High school and below | ref |  | ref |  |
| College /undergraduate | 2.025 (1.302-3.151) | **0.002** | 0.982 (0.540-1.789) | 0.954 |
| Master’s degree and above | 1.012 (0.471-2.178) | 0.975 | 0.356 (0.134-0.945) | **0.038** |
| **Average monthly household income** |  |  |  |  |
| <3000 | ref |  | ref |  |
| 3000-5000 | 0.585 (0.345-0.993) | **0.047** | 0.682 (0.368-1.263) | 0.223 |
| 5000-10000 | 0.362 (0.193-0.680) | **0.002** | 0.455 (0.216-0.957) | **0.038** |
| >10000 | 1.794 (0.803-4.006) | 0.154 | 3.356 (1.222-9.214) | **0.019** |
| Prefer not to disclose | 0.846 (0.520-1.375) | 0.499 | 1.470 (0.835-2.587) | 0.182 |
